# Twenty-four weeks of combined exercise training prevents metabolic syndrome progression in adult women: evidence from a randomized controlled trial

**DOI:** 10.5114/biolsport.2026.153313

**Published:** 2025-08-13

**Authors:** Mouna Abrougui, Refka Hassine, Monia Zaouali, Asma Ben Abdelaziz, Asma Omezzine, Meriam Denguezli

**Affiliations:** 1 Department of Basic Sciences, LR19ES09, Faculty of Medicine of Sousse, University of Sousse, Sousse, Tunisia; 2 Department of Biochemistry, LR12SP11, Sahloul University Hospital, Sousse Tunisia. Faculty of pharmacy of Monastir, University of Monastir, Monastir, Tunisia; 3 Department of Basic and Mixed Sciences, Faculty of Dental Medicine, University of Monastir, Monastir, Tunisia

**Keywords:** Combined exercise training, Metabolic syndrome, Glycaemic control, Glycolipid metabolism, Functional fitness, Cardiovascular risk factors

## Abstract

Metabolic syndrome (MetS) is a cluster of key clinical risk factors for cardiovascular disease (CVD) and type 2 diabetes mellitus (T2DM), making it essential to address its components through targeted lifestyle interventions, such as exercise. This study aimed to investigate the impact of a combined training (CT) programme on adult women diagnosed with MetS. We hypothesized that participating in the programme would improve glucose and lipid metabolism, cardiovascular health, functional fitness abilities (FFA), body composition and anthropometrics in women with MetS. 105 inactive women were randomly assigned to either a CT (n=84) or control group (n=21). The CT group performed supervised combined aerobic and resistance training 3 days/week for 6 months. Blood glucose, haemoglobin A1c (HbA1c), insulin levels, homeostatic model assessment for insulin resistance (HOMA-IR), lipid profile, mean arterial pressure (MAP), resting (r-HR) and maximal heart rate (max-HR), body composition, anthropometrics and FFA were assessed before and after 3 and 6 months of training. After 3 months, the CT group showed significant improvements compared to controls in blood glucose (-36.4%), HbA1c (-22.8%), HOMA-IR (-12.5%), MAP (-16.9%), max-HR (-5.3%), r-HR (-20.4%), skeletal muscle to fat ratio (MFR) (+37.5%), and FFA (+200%). At 6 months, benefits were sustained or enhanced, particularly in glycaemic regulation, cardiovascular parameters, MFR, and FFA levels (p < 0.05). MFR was positively and linearly associated with changes in FFA, cardiovascular function, and glycometabolic markers. A 24-week CT programme significantly improved key MetS parameters in women, suggesting that it may be an effective non-pharmacological strategy to reduce CVD and T2DM risk.

## INTRODUCTION

Metabolic syndrome (MetS) is a major risk factor for increased morbidity and mortality, particularly with aging. It significantly contributes to the development of cardiovascular disease (CVD) and diabetes, as well as other chronic conditions, including osteoarthritis, liver and kidney disease, sleep apnoea, and depression [1].

The prevalence of MetS increased dramatically worldwide, reaching epidemic levels, particularly in less developed nations [1]. This condition imposes substantial social and economic burdens, underscoring the urgent need for effective strategies to reduce healthcare costs. Based on existing evidence, interventions that effectively manage both blood glucose and cholesterol levels are highly promising in the prevention of CVD. The global fitness industry is evolving, with a growing focus on exercise as a vital pillar of overall health and well-being, rather than merely a recreational pastime [2]. A personalized approach, which considers the specific type of activity, is essential for optimal results [3]. Consequently, consistent engagement in appropriate exercise programmes can significantly promote better health outcomes [4]. The latest recommendations for managing adult obesity emphasize the necessity of regular multimodal exercise to enhance cardiometabolic health [5].

Numerous studies have explored the impact of aerobic exercise on various health outcomes [6]. For effective weight loss and improvements in cardiometabolic health, consistent endurance training is crucial [7]. During aerobic workouts, the body primarily utilizes fat for energy, enhancing its ability to break down fat stores (lipolysis) [8]. The American College of Sports Medicine and the American Heart Association recommend that adults engage in at least 150 minutes of moderate or 75 minutes of vigorous aerobic exercise weekly to maintain overall health [9].

Resistance training has been shown to enhance glucose consumption by stimulating muscle hypertrophy and shifting muscle fibre types [10]. It aids in weight management and improves insulin sensitivity, lipid profiles, and glycaemic control [11]. High-intensity resistance workouts primarily burn carbohydrates for immediate energy and stimulate the release of hormones such as growth hormone and testosterone. These hormones play a vital role in muscle growth and enhance the body’s capacity to access glucose from fat stores through lipolysis [12]. The beneficial effects of resistance training are attributed to increased muscle mass, a higher number of insulin receptors in muscle cells, and an elevated presence of glucose transporter proteins [7].

A combined exercise regimen that integrates both aerobic and resistance training within a single session has been shown to positively influence body mass and composition, while also improving glucose and lipid metabolism [13]. According to current American Diabetes Association guidelines, incorporating both aerobic and resistance exercises is considered the most effective strategy for managing lipid profiles and glucose levels [14]. Moreover, compared to standard pharmacological interventions, the combination of aerobic and resistance training has been shown to be a cost-effective strategy for optimally combating MetS. Indeed, the use of such training programmes could reduce the economic burden not only for the individual but also for insurance companies, thus providing a muchneeded service to public health [15].

Despite the elevated prevalence of MetS, research on the impact of resistance and aerobic exercise training on risk factors related to the glycolipid profile is limited, with even fewer studies examining these training types in combination. Further investigations are needed to understand how combined training (CT) influences these risk factors.

This study aimed to evaluate the effect of a 24-week combined aerobic and resistance training programme on MetS-related risk factors, including cardiovascular health, glucose and lipid metabolism, functional fitness ability (FFA), body composition, and anthropometric measures in adult women with MetS.

We hypothesized that participating in the programme would lead to improvements in MetS-related risk factors.

## MATERIALS AND METHODS

### Design and protocol registration

This study has been reported in accordance with the CONSORT guidelines. It incorporated a repeated measures component to track within-subject changes over time, while also using randomized control and experimental groups to evaluate differences between groups.

To ensure ethical conduct, participants were completely told about all procedures, hazards and protocols before obtaining their informed written consent. This adhered to the ethical guidelines established by the American College of Sports Medicine [16]. Additionally, the study procedures were reviewed and approved by the Ethics Committee of Sousse Medical University (CEFMS 160/2023). Furthermore, a comprehensive protocol governing our study procedures has been formally recorded on the International Clinical Trials Registry Platform (ClinicalTrials.gov), and it is identified with the project number PACTR202501917477092.

Three sets of measurements were conducted at distinct time points: (1) *baseline*, before the intervention; (2) *mid-intervention*, at the end of week 12; and (3) *post-intervention*, 48–72 h after the conclusion of 24-week training. All data collection and testing were conducted between 8:30 and 10:00 AM to minimize diurnal variations. Measurements were taken twice by the same researcher, while tests and laboratory analyses were performed by investigators blinded to group assignment. To reduce potential nutritional bias, participants were encouraged to maintain their habitual dietary intake throughout the study.

In each session, participants underwent a three-day assessment process within the same week:

*Day 1: Participants screening.* All the subjects underwent a medical history assessment using the Physical Activity Readiness Questionnaire [17]. Systolic (SBP) and diastolic blood pressures (DBP), anthropometric measurements and body composition were recorded, and blood samples were collected for serum and plasma analysis.

*Day 2:* The participants underwent a muscular strength test to determine the appropriate training intensity for the resistance training protocol by assessing their one-repetition maximum (1RM). Additionally, functional fitness tests were performed to assess FFA.

*Day 3:* Participants underwent a maximal graded exercise to determine their aerobic power.

### Inclusion criteria

(a) Having a MetS as defined according to the 2009 International Diabetes Federation (IDF) standards [18]; (b) aged ≥ 35 years; (c) physically inactive – defined as engaging in < 1 hour of exercise regularly per week within the previous year and, (d) were deemed capable of performing the required exercises.

### Non-inclusion criteria

(a) absolute or relative physical exercise counter-indication, (b) any cardiovascular, respiratory, or musculoskeletal conditions that would prevent participation in physical exercise, (c) a resting heart rate (r-HR) > 120 bpm, SBP > 180 mmHg and/or DBP > 100 mmHg, (d) pregnancy or menstrual cycle instability, (e) smoking or alcohol consumption, (f) any change in the subjects’ routine programme based on a medical opinion, and (g) the use of corticosteroids, oral contraceptives, or adherence to a specific dietary regimen.

### Sample size

The optimal sample size for this study was determined using the G*Power program. Based on the methodology outlined by Santoro et al. [19], a sample size of 130 participants was calculated. The calculation considered a significance level (α) of 0.05 and a statistical power of 0.8. To account for a 10% nonresponse rate, the final required sample size was set at 143 participants. This ensured that the intended statistical power and significance level were met for detecting the expected effect size.

### Participants

**FIG. 1:**
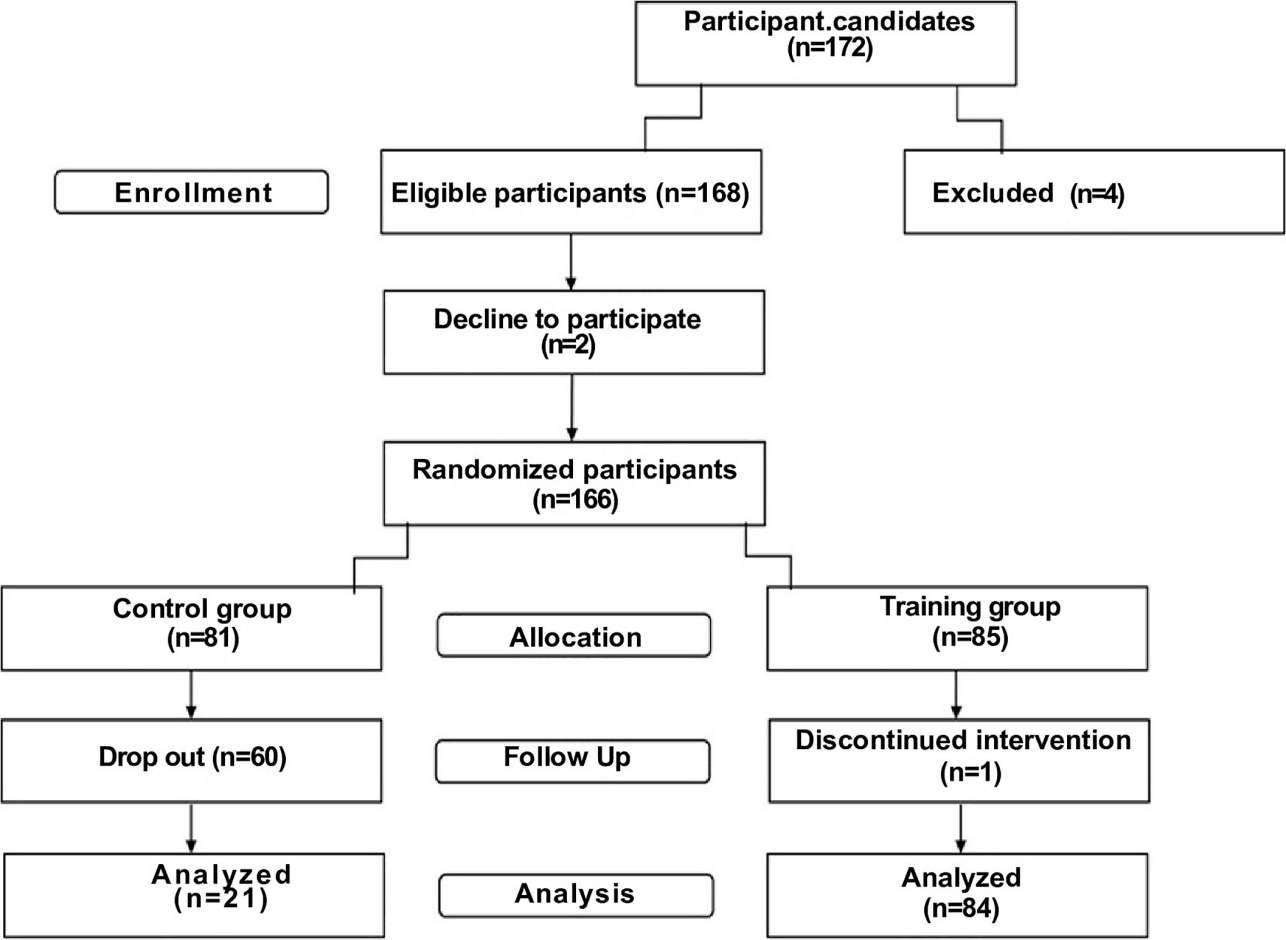
Flowchart of participant recruitment.

After pre-measurements, a final sample of 166 individuals with MetS were included in the present study. These participants were randomly assigned to either a control group or a CT group. Over time, 60 participants from the control group withdrew, while 84 from the CT group successfully completed the protocol and were included in the final analysis (Figure 1).

### Biochemical study

Haemoglobin A1c (HbA1c), insulin levels, and homeostatic model assessment for insulin resistance (HOMA-IR) were measured. HbA1c level was determined from whole blood in EDTA-containing tubes. The blood glucose level and blood lipid profiles, including triglycerides (TG), total cholesterol (TC), low-density lipoprotein cholesterol (LDLC), and high-density lipoprotein cholesterol (HDL-C), were assessed using a spectrophotometric enzymatic method on a UnicelDxC 660i automatic biochemistry analyser (Beckmann Coulter, Fullerton, CA, USA). Insulin concentration was measured using an electrochemiluminescence immunoassay (ECLIA) on the Roche Cobas E411 analyser. Insulin resistance was determined by the homeostatic model assessment for insulin resistance (HOMA-IR) calculated as described by Matthews et al. [20].

### Functional fitness tests

The functional fitness assessments in this study evaluate physical attributes through tasks involving functional movements [21]. These assessments comprised six straightforward tasks designed to evaluate aerobic capacity, muscular strength, flexibility and postural control. Specifically, the trunk flexion test was used to assess the flexibility of the lower back and hamstrings, while the 60-second single-leg balance test measured static balance and postural control. The 30-second chair stand test evaluated lower-body strength, and the arm curl test evaluated upper-body strength. The scapulo-humeral mobility test was used to evaluate shoulder joint mobility and muscular function, and the 6-minute walk test measured aerobic endurance [21]. Each test was performed twice, and the highest score was recorded. A global functional fitness score ranging from 0 to 30 was calculated by summing the scores from the six standardized physical fitness tests. Each test was scored from 1 to 5 based on age- and sex-specific normative percentiles derived from reference populations [21, 22].

### Aerobic power

Participants’ ${\dot{\text{V}}\text{O}}_{2\max}$ was estimated following the modified Bruce protocol. The treadmill test commenced at a speed of 2.74 km/h (1.7 mph) with no incline (BT Fitness, 360+, Alicante, Spain). At three-minute intervals, either the speed, the incline, or both were progressively increased. The test was concluded when participants could no longer continue due to exhaustion, discomfort, or other medical reasons, as previously outlined. ${\dot{\text{V}}\text{O}}_{2\max}$ was then predicted using the following formula: ${\dot{\text{V}}\text{O}}_{2\max}$ = 14.8 – (1.379 × T) + (0.451 × T^2^) – (0.012 × T^3^), where T is the time (min) to complete the test [23].

### Muscular strength testing

Before each testing phase, the appropriate intensity for the resistance training protocol was determined using the 1RM assessment. Following a brief warm-up, strength testing was conducted for all exercises included in the RT programme. Each participant completed two attempts with a 5-minute rest interval between them, and the highest number of repetitions performed was recorded. The maximal strength was estimated using a previously established formula: 1RM = weight / (1.0278 − 0.0278 × reps) [24].

### Combined training intervention

**FIG. 2:**
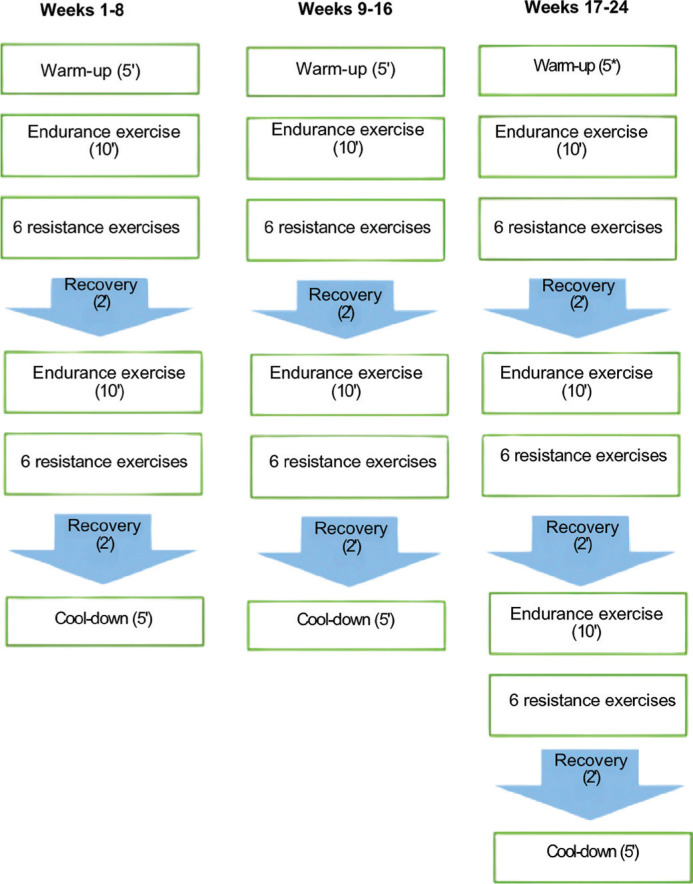
Training protocol

The exercise regimen for older adults in the CT group consisted of structured endurance training followed by resistance training sessions. These sessions took place on Mondays, Wednesdays, and Fridays, lasting 60 minutes per session, over a period of 24 weeks. Participants trained in small groups of 5–6 individuals. If a participant was unable to attend a session for any reason, individualized supplementary training was arranged to ensure continuity. During each session participants started with a 5-minute warm-up, proceeded with 50 minutes of main training, and finished with a 5-minute cool-down (Figure 2).

### Aerobic training protocol

The aerobic training protocol started with 20 minutes at 50% of maximum heart rate on a treadmill (BT Fitness, 360+, Alicante, Spain) from week 1 to 8. Over the course of the intervention, the duration and intensity were gradually increased, reaching 30 minutes at 70% of maximal heart rate (max-HR) in the final period. The intensity of exercise was objectively tracked using a Polar HR monitor (Polar H9; Polar Electro, France).

### Resistance training protocol

The resistance training began at 40% of the 1RM and progressively increased to 70% by the final week of the programme. During weeks 1 to 8, participants completed 2 sets with 8 to 16 repetitions per set at 40–50% of their 1RM with 2 minutes of rest between sets, for a total of 20 min [25]. From weeks 9 to 16, the repetitions increased to 16 to 24 per set, with the intensity rising to 50–60% of 1RM. In weeks 17 to 24, they completed 3 sets with 24 to 32 repetitions per set at 60–70% of 1RM. The exercise routine consisted of shoulder flexions, squats, bicep curls, triceps extensions, single-leg balance drills, knee extensions and flexions, side dips, abdominal crunches, static planks, side leg raises, modified scissors, and glute bridges. The general training intervention approach is summarized in Table 1.

**TABLE 1:** Training details

Weeks	Intensity		Duration	

	ET (HR_max_)	RT (1RM)	ET minutes)	RT (repetitions)
Weeks 1 to 8	50%	40%–50%	20	8–16
Weeks 9 to 16	60%	50%–60%	20	16–24
Weeks 17 to 24	70%	60%–70%	30	24–32

ET, Endurance training; RT, Resistance training; HR, Heart rate; 1RM, One repetition maximum.

### Statistical analysis

All statistical analyses were performed using IBM SPSS Statistics software (version 23). The Kolmogorov–Smirnov test was used to assess the normality of continuous variables. Data following a normal distribution are presented as mean ± standard deviation, while non-normally distributed data are expressed as median and interquartile range. Variables with a non-Gaussian distribution were logtransformed prior to analysis. A mixed-factorial ANOVA was conducted to evaluate the effects of time, group, and their interaction on the various outcome measures. To control for multiple comparisons, the Bonferroni correction was applied. Effect sizes were reported using partial eta squared (η^2^_p_), with thresholds interpreted as follows: < 0.01 (small), > 0.06 (moderate), and > 0.14 (large) [26]. Odds ratios and corresponding 95% confidence intervals were calculated using multivariable logistic regression models. Participants with incomplete data at any time point (T0, T1, or T2) were excluded from the analyses; consequently, all statistical tests were conducted on complete cases without any imputation for missing data. A p-value of < 0.05 was considered statistically significant.

## RESULTS

The CT programme was well tolerated, with no participants reporting any adverse events or negative symptoms throughout the intervention. The CT group exhibited 100% adherence to the supervised CT programme, whereas several dropouts occurred in the control group. Baseline measurements did not differ significantly between groups (p > 0.05; Table 2).

**TABLE 2:** Physiological and anthropometric parameters in three stages (Baseline, 12 weeks, 24 weeks)

Characteristics	Control group (n=21; 20%)			Trained group (n=84; 80%)				

	T0 – baseline	T1 – 12 weeks	T2 – 24 weeks	T0 – baseline	T1 – 12 weeks	T2 – 24 weeks	Time effect	Interaction effect
Age, years	55.8 ± 7.3	–	–	52.8 ± 7.9	–	–		

Weight, Kg	78.7 ± 10.0	79.6 ± 9.9	81.4 ± 10.6 ^§^	83.4 ± 14.2	79.4 ± 10.9 ^£^	74.1 ± 9.6^§Ұ^	0.705	< 0.001

Height, cm	162.4 ± 6.6	–	–	161.2 ± 6.2	–	–		

BMI, Kg/m^2^	30.8 ± 4.0	31.2 ± 3.9 ^£^	31.9 ± 4.6^§^	32.3 ± 4.7	30.7 ± 4.6^£^	28.6 ± 4.3^§Ұ^	0.492	< 0.001

Waist circumference, cm ^a^	103.1 (100.0–115.0)	106.5 (101.2–113.1)	107.3 (105.1–115.2)	107.7 (102.0–116.8)	99.9 (93.1–105.4) ^£^	90.6 (82.0–98.1) ^§Ұ^	0.001	< 0.001

Hip circumference, cm^a^	111.1 (102.5–117.5)	110.1 (105.0–118.5)	112.2 (106.5–120.4)	113.1 (107.3–122.1)	107.4 (100.1–114.9) ^£^	99.2 (92.1–106.3) ^§Ұ^	0.168	< 0.001

WHR^a^	0.97 (0.92–1.05)	0.96 (0.93–1.03)	0.96 (0.95–0.99)	0.94 (0.90–0.99)	0.92 (0.87–0.98) ^£^	0.88 (0.84–0.95) ^§Ұ^	0.011	0.023

Total fat, %^a^	34.9 (30.1–38.2)	34.5 (33.1–38.2)	37.9 (34.1–39.6)	36.1 (33.0–38.7)	30.3 (29.1–33.5) ^£^	26.7 (25.6–28.7) ^§Ұ^	< 0.001	< 0.001

Skeletal muscle, %^a^	27.1 (24.6–30.7)	25.2 (24.2–28.3)	23.1 (22.0–26.3) ^§^	24.5 (22.2–28.6)	30.0 (28.6–32.0) ^£^	33.1 (32.0–34.2) ^§Ұ^	< 0.001	< 0.001

Visceral fat, %^a^	7.0 (6.7–7.3)	7.5 (7.0–7.9)	8.1 (7.9–8.3)	7.3 (6.7–8.3)	6.5 (6.0–7.3) ^£^	5.9 (5.0–6.4) ^§Ұ^	0.001	< 0.001

MFR^a^	0.77 (0.67–1.01)	0.72 (0.66–0.86) ^£^	0.60 (0.57–0.74) ^Ұ^	0.66 (0.58–0.79)	0.99 (0.87–1.08) ^£^	1.24 (1.10–1.33) ^§Ұ^	< 0.001	< 0.001

Systolic blood pressure, mmHg	146.4 ± 10.6	152.6 ± 11.9	157.8 ± 12.5^§^	147.9 ± 11.3	126.4 ± 14.7^£^	120.3 ± 12.3^§ Ұ^	< 0.001	< 0.001

Diastolic blood pressure, mmHg	91.2 ± 3.8	93.1 ± 6.7	91.8 ± 7.4	92.2 ± 4.2	77.5 ± 6.4 ^£^	74.6 ± 4.9 ^§Ұ^	< 0.001	< 0.001

Mean arterial pressure, mmHg	109.6 ± 5.4	112.9 ± 6.9	113.8 ± 7.5^§^	110.8 ± 5.7	93.8 ± 7.6^£^	88.8 ± 11.5^§Ұ^	< 0.001	< 0.001

HR at rest, c/min^a^	78.0 (70.0–82.0)	84.0 (78.0–88.0) ^£^	88.0 (82.0–89.0) ^§^	76.0 (69.0–81.0)	72.0 (68.0–76.0) ^£^	70.0 (67.0–73.0) ^§Ұ^	< 0.001	< 0.001

HR max, c/min^a^	129.0 (125.0–137.0)	138.0 (133.0–143.0) ^£^	144.0 (139.0–145.0) ^§Ұ^	132.0 (128.0–138.0)	125.0 (120.0–126.0) ^£^	110.0 (108.0–120.0) ^§Ұ^	< 0.001	< 0.001

BMI, body mass index; WHR, waist to hip ratio; MFR, Skeletal muscle to fat ratio; HR, heart rate;

£ , Significant difference between T0 and T1 within each group (p < 0.01);

§ , Significant difference between T0 and T2 within each group;

Ұ , Significant difference between T1 and T2 within each group;

a , Logarithm-transformed values were used for comparison.

Over the 24-week period, significant time × group interactions were observed for body weight, BMI, waist and hip circumferences, waist-to-hip ratio (WHR), total and visceral fat, skeletal muscle percentage, and muscle-to-fat ratio (MFR) (all p < 0.001, except WHR: p = 0.023). Participants in the CT group showed significant reductions in BMI, waist circumference, and fat mass, along with increases in skeletal muscle and MFR (all p < 0.001), while the control group exhibited opposite trends. Repeated-measures ANOVA confirmed large effects for all anthropometric parameters. Two key examples are highlighted: BMI (time: F = 23.29, η^2^_p_ = 0.184; group: F = 273.30, η^2^_p_ = 0.728; interaction: F = 85.68, η^2^_p_ = 0.454) and MFR (interaction: F = 110.12, η^2^_p_ = 0.517).

Repeated-measures ANOVA revealed significant time × group interaction effects for SBP, DBP, mean arterial pressure (MAP), r-HR and max-HR over the 24-week intervention, (all *p* < 0.001). In the CT group, both SBP and DBP decreased progressively over time, whereas the control group exhibited a trend toward deterioration. MAP followed a similar pattern, with significant main effects of time (*F* = 27.05, *p* < 0.001, η^2^_p_ = 0.208) and group (*F* = 106.24, *p* < 0.001, η^2^_p_ = 0.508), and a robust time × group interaction (*F* = 59.26, *p* < 0.001, η^2^_p_ = 0.365), indicating clinically meaningful reductions in the CT group.

R-HR decreased significantly in the CT group, while increasing in the control group, with a strong interaction effect (*F* = 42.62, *p* < 0.001, η^2^_p_ = 0.295). Notably, the main effect of time was not significant (*p* = 0.143), suggesting differential group trajectories.

The CT group also exhibited a marked reduction in max-HR, in contrast to an increase in the control group. This was supported by large main effects of time (*F* = 28.67, *p* < 0.001, η^2^_p_ = 0.223) and group (*F* = 61.88, *p* < 0.001, η^2^_p_ = 0.382), and an especially strong time × group interaction (*F* = 230.78, *p* < 0.001, η^2^_p_ = 0.698) (Table 2).

No substantial changes were observed over the 24-week period in HDL-C levels in either group. For LDL-C, a significant time effect was detected (*F* = 9.11, *p* < 0.001, η^2^_p_ = 0.081), while the time × group interaction showed a borderline effect (*F* = 3.14, *p* = 0.050, η^2^_p_ = 0.030), indicating a modest reduction in LDL-C in the CT group. Although TC showed no main effects, a significant interaction was found (F = 5.27, p = 0.011, η^2^_p_ = 0.049), with levels declining in the CT group. TG levels also decreased slightly, but changes were not statistically significant (p > 0.05). Neither the main effect for time (*p* = 0.292) or group (*p* = 0.629) nor the interaction (*p* = 0.078) was statistically significant (Table 3).

**TABLE 3:** Lipid profile and glucose metabolism in three stages (Baseline, 12 weeks, 24 weeks).

Characteristics	Control group (n=21, 20%)			Trained group (n=84, 80%)				

	T0 – Baseline	T1 – 12 weeks	T2 – 24 weeks	T0 – Baseline	T1 – 12 weeks	T2 – 24 weeks	Time effect	Interaction effect
HDL-C, mmol/L ^a^	1.3 (1.2–1.5)	1.4 (1.3–1.8)	1.3 (1.1–1.5)^Ұ^	1.3 (1.1–1.5)	1.4 (1.2–1.6)	1.3 (1.2–1.5) ^Ұ^	0.361	0.239
LDL-C, mmol/L ^a^	3.5 (2.8–4.1)	3.2 (2.7–4.1)	3.4 (2.3–4.2)	3.6 (3.1–4.6)	3.1 (2.6–3.9) ^£^	3.2 (2.9–3.9) ^§^	0.001	0.050
TC, mmol/L ^a^	5.4 (4.7–6.2)	5.6 (4.8–6.5)	5.6 (4.4–6.5)	5.6 (4.9–6.7)	5.1 (4.4–6.0) ^£^	5.2 (4.8–5.9) ^§^	0.541	0.011
TG, mmol/L ^a^	1.3 (0.95–1.75)	1.2 (0.9–1.8)	1.4 (0.8–1.9)	1.4 (1.0–1.8)	1.2 (1.0–1. 6) ^£^	1.1 (0.9–1.6) ^§^	0.292	0.078
Glucose, mmol/L	8.5 ± 3.3	9.6 ± 5.3	9.6 ± 5.5	7.9 ± 2.6	6.1 ± 1.2 ^£^	6.1 ± 1.4 ^§^	<0.001	<0.001
HbA1c, %	7.8 ± 2.1	7.9 ± 2.2	7.8 ± 2.2	7.6 ± 1.5	6.1 ± 1.0 ^£^	6.1 ± 0.9 ^§^	<0.001	0.006
Insulin, mU/ml ^a^	7.9 (5.4–14.2)	7.8 (5.6–11.4)	7.4 (5.5–13.1)	8.1 (6.4–14.6)	7.4 (5.7–12.1) ^£^	7.1 (5.2–13.8) ^§ Ұ^	0.004	0.216
HOMA-IR ^a^	2.8 (1.5–6.4)	2.4 (1.5–5.5)	3.7 (1.9–5.6)	3.1 (2.2–4.7)	2.1 (1.4–3.5) ^£^	2.5 (1.7–4.1) ^§ Ұ^	0.009	0.019

HDL-C, High density lipoprotein-cholesterol; LDL-C, Low density lipoprotein-cholesterol; TG, Triglyceride; TC, Total cholesterol; HbA1C, glycated haemoglobin; HOMA-IR, Homeostatic Model Assessment for Insulin Resistance;

£ , Significant difference between T0 and T1 within each group;

§ , Significant difference between T0 and T2 within each group;

Ұ , Significant difference between T1 and T2 within each group;

a , Logarithm-transformed values were used for comparison.

Over time, the CT group showed marked improvements in fasting glucose and HbA1c, while levels remained elevated in controls. A significant time × group interaction was found for glucose (*F* = 9.35, *p* < 0.001, η^2^_p_ = 0.083) and HbA1c (F = 5.27, p = 0.006, η^2^_p_ = 0.049) indicating a greater reduction in the CT group compared to no improvement in the control group. For HOMA-IR, both a main effect of time (*F* = 4.82, *p* = 0.009, η^2^_p_ = 0.049) and a significant time × group interaction (*F* = 4.04, *p* = 0.019, η^2^_p_ = 0.041) were observed, supporting improved insulin sensitivity in the CT group. Although insulin levels showed a significant main effect of time (*F* = 5.72, *p* = 0.004, η^2^_p_ = 0.057), the time × group interaction was not significant (*p* = 0.216), suggesting parallel reductions in both groups (Table 3).

After 12 and 24 weeks, the CT group exhibited marked improvements across all functional fitness tests (all *p* < 0.001), whereas performance in the control group remained stable or declined. The global functional fitness score increased significantly in the CT group (time × group interaction: F = 186.06, p < 0.001, η^2^_p_ = 0.650), reflecting substantial gains in physical performance (Table 4).

**TABLE 4:** Physical fitness data in three stages (Baseline, 12 weeks, 24 weeks).

	Control group (n=21; 20%)			Trained group (n = 84; 80%)		

Characteristics	T0 – baseline	T1 – 12 weeks	T2 – 24 weeks	T0 – baseline	T1 – 12 weeks	T2 – 24 weeks	Time effect	Interaction effect
One leg balance test, s^a^	2.3 (1.3–4.1)	2.2 (1.1–5.5)	1.1 (0.9–3.1)	2.9 (1.4–7.1)	20.1 (14.3–30.0) ^£^	35.0 (27.3–40.0) ^§Ұ^	< 0.001	< 0.001

Trunk flexion, score ^a^	1.0 (1.0–1.0)	1.0 (1.0–1.0)	1.0 (1.0–1.0)	1.0 (1.0–1.0)	3.0 (2.0–3.0) ^£^	4.0 (4.0–5.0) ^§ Ұ^	< 0.001	< 0.001

Sitting handgrip, rep ^a^	33.0 (27.5–34.5)	29.0 (26.0–32.2)	26.1 (23.5–30.3)	33.0 (29.6–34.1)	52.0 (48.0–56.0) ^£^	70.0 (65.0–76.0) ^§Ұ^	< 0.001	< 0.001

Scapulo humeral mobility, score ^a^	1.0 (1.0–1.0)	1.0 (1.0–1.0)	1.0 (1.0–1.0)	1.0 (1.0–1.0)	2.0 (2.0–2.0) ^£^	4.0 (3.0–4.0) ^§Ұ^	< 0.001	< 0.001

30-s chair stand test, rep ^a^	13.0 (12.0–15.0)	13.0 (10.0–14.0)	13.0 (10.0–14.0)	13.0 (11.0–15.0)	25.0 (21.0–26.0) ^£^	32.0 (30.0–34.0) ^§Ұ^	< 0.001	< 0.001

6-min walk test, m ^a^	410.2 (325.3–435.6)	302.4 (300.0–361.2)	304.9 (301.5–312.7)	368.6 (306.2–422.5)	541.8 (481.4–597.9) ^£^	661.1 (601.2–721.3) ^§Ұ^	< 0.001	< 0.001

Functional fitness s, score ^a^	6.0 (6.0–7.0)	6.0 (6.0–6.0)	6.0 (6.0–6.0)	6.0 (6.0–7.0)	18.0 (17.0–19.0) ^£^	25.0 (24.0–26.0) ^§Ұ^	< 0.001	< 0.001

Rep, repetition;

£ , Significant difference between T0 and T1 within each group;

§ , Significant difference between T0 and T2 within each group;

Ұ , Significant difference between T1 and T2 within each group;

a , Logarithm-transformed values were used for comparison.

**FIG. 3:**
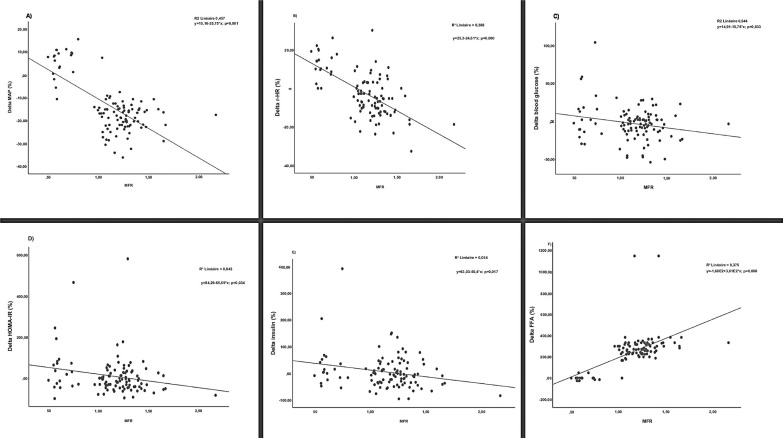
Linear regressions of delta mean arterial pressure (MAP), resting heart rate (r-HR), blood glucose, insulin resistance (HOMA-IR), insulin and functional fitness ability (FFA) as a function of skeletal muscle to fat ratio (MFR).

Furthermore, a significant and direct linear association was observed between the increase in MFR and improvements in multiple metabolic and cardiovascular parameters. Specifically, elevated MFR was strongly correlated with improvements in FFA (OR = 361.342; 95%CI = 268.801, 453.884; p < 0.001), MAP (OR = -25.753; 95%CI = -31.243, -20.263; p = 0.001), r-HR (OR = -24.614; 95%CI = -30.684, -18.543; p < 0.001), blood glucose (OR = -15.781; 95%CI = -30.233, -1.328; p = 0.033), insulin (OR = -50.400; 95%CI = -91.631, -9.169; p = 0.017) and HOMA-IR (OR = -65.688; 95%CI = -126.164, -5.211; p = 0.034) (Figure 3).

## DISCUSSION

The aim of this study was to investigate the effects of a 24-week CT programme on glycolipid metabolism, cardiovascular health, FFA, body composition and anthropometrics in inactive adult women with MetS. The findings provide several novel insights into the current literature:

1. *Early efficacy of CT*: Just 12 weeks of CT significantly improved glucose metabolism, cardiovascular health, body composition, and FFA of women with MetS.
2. *Sustained benefits over time*: Benefits in cardiovascular function, body composition, and FFA were either maintained or further enhanced after 24 weeks, demonstrating lasting physiological adaptations.
3. *MFR as a biomarker*: Improvements in MFR are associated with gains in cardiovascular function, glucose regulation, and physical performance, supporting its utility for monitoring exercise interventions in women with MetS.
4. *Limited impact on lipid profile*: A 24-week CT programme was insufficient to significantly improve lipidic markers, suggesting that longer or more intensive interventions may be required.

These findings indicate that CT can be an effective approach for improving MetS risk factors. Following the exercise intervention, participants exhibited a notable increase in lean body mass, accompanied by significant reductions in body fat percentage and central adiposity, culminating in an improved MFR. These findings are consistent with previous studies demonstrating that CT in obese middle-aged women effectively reduces body weight, body fat percentage, and WHR [27]. Our findings also challenge the notion that caloric restriction must be a central component of exercise strategies aimed at reducing abdominal obesity and associated risk factors in adults [28]. Indeed, despite the modest 11.1% weight loss observed in the CT group, visceral fat was reduced by approximately 19%, total fat was reduced by 26%, and skeletal muscle increased by 35%. These results have important implications for individuals who find substantial weight loss difficult to achieve, as altering lifelong habits related to nutrition and exercise can often lead to distress and anxiety [29].

Our results demonstrated significant improvements in glucose metabolism markers in women with MetS after both 12 and 24 weeks of CT. Notably, fasting glucose, HbA1c and insulin resistance were all significantly reduced. These findings highlight the short-term efficacy of CT, with just 12 weeks leading to substantial metabolic improvements compared to untrained controls. This rapid physiological response is particularly noteworthy in women, a population underrepresented in previous research. In line with our findings, Philipsen et al. also reported comparable improvements in individuals who experienced reductions in both visceral and total fat mass following exercise training [30]. Sarafidis and Bakris further emphasized that visceral fat loss enhances peripheral glucose uptake while suppressing hepatic glucose production [31]. This is supported by the significant association we observed between muscle-to-fat ratio and training-improved glucose metabolism in our patients with metabolic syndrome (Figure 3C). These anthropometric changes likely played a key role in driving the more favourable metabolic profile documented in our study.

However, our results revealed no statistically significant difference in insulin levels between groups. Hersey et al. conducted a 6-month randomized controlled trial and found that aerobic exercise led to significant reductions in fasting insulin levels [32]. A recent metaanalysis supports these findings, indicating that CT was less effective in improving insulin levels than aerobic training alone [4]. Nevertheless, the strong association observed in our study between MFR and training-induced changes in insulin (Figure 3E) suggests that greater improvements in body composition, potentially achieved through longer or more intensive training, could lead to more pronounced benefits in insulin regulation.

Despite the known beneficial effects of exercise on lipid metabolism, TG levels in our study remained unchanged after 24 weeks of CT (180 min/week). This may reflect insufficient training stimulus, low baseline TG levels (1.4 mmol/L), or individual variability in metabolic response. Similar null results were reported by Park et al. [33] and Ha and So [34] after 12-week CT interventions, while Ahn and Kim [25] observed TG reductions following six months of dynamic resistance training at a lower weekly volume, suggesting that intervention duration and participant characteristics may outweigh training volume alone. Likewise, HDL-C levels did not improve, in line with prior studies showing that moderateintensity or short-duration programmes are often inadequate for eliciting HDL-C gains in metabolically compromised populations [3]. Mechanisms such as enhanced reverse cholesterol transport and enzyme modulation may require more intensive or prolonged interventions, possibly combined with dietary changes [35]. A significant time effect for LDL-C (p < 0.001) and a borderline time × group interaction (p = 0.050) suggest modest LDL-C reductions attributable to the CT programme. Previous studies have shown stronger effects with high-intensity or higher-volume training [7, 36]. Total cholesterol did not show a main effect but decreased significantly in the CT group (interaction p = 0.011), reinforcing the potential for CT to improve lipid profiles.

Our findings revealed a significant association between improvements in MFR and reductions in MAP, highlighting the role of body composition in cardiovascular regulation (Figure 3A). This may be due to reduced fat mass lowering pro-inflammatory adipokines, increased lean mass protecting against hypertension [37, 38], and enhanced endothelial function via improved nitric oxide (NO) availability. Similar benefits have been reported with higher-intensity activities such as recreational soccer or basketball in overweight individuals [39]. Additionally, exercise-induced vasodilation – mediated largely by NO and supported by HSP90 pathways –may further contribute to blood pressure improvements [40]. Moreover, the observed reduction in r-HR is a well-established marker of improved autonomic regulation and cardiovascular efficiency, often associated with increased stroke volume and enhanced parasympathetic tone [40]. Similarly, max-HR showed a marked reduction in the CT group compared to an increase in controls. These findings reflect enhanced cardiovascular efficiency and training-induced adaptations such as improved oxygen delivery, reduced cardiac workload at submaximal effort, and possibly an improved chronotropic response. This effect may also be attributed to exercise-induced release of anti-inflammatory and cardioprotective molecules. For example, galectin-3, elevated in endurance athletes, supports immune activation and offers potential protection against cardiovascular damage. Likewise, cytokines such as IL-10 and the IL-33/ST2 axis contribute to reduced cardiac fibrosis and enhanced myocardial function, thereby strengthening cardiac resilience during and after exercise [40].

Our research study provides evidence that FFA of the women with MetS is substantially improved in response to 12- and 24-week CT. This aligns with previous research demonstrating greater functional gains from combined aerobic and resistance exercise compared to either modality alone [4]. Cross-sectional evidence supports MFR as a strong predictor of physical function, with higher ratios linked to faster gait speed and fewer limitations [34]. Our results build on this by showing a significant positive association between MFR and FFA scores (Figure 3F), underscoring the role of body composition in functional capacity.

The CT model used in this study could be translated into community and public health programmes targeting women with metabolic syndrome. Its demonstrated efficacy within 12 weeks, straightforward structure, and use of accessible exercises support its implementation in various settings such as community health centres, local organizations, and workplace wellness initiatives. Group sessions supervised by qualified professionals may enhance adherence through social support while minimizing costs. Scaling this model could offer a practical strategy to improve cardiometabolic health in at-risk women through preventive, population-level interventions.

## CONCLUSIONS

Our findings demonstrate that CT significantly improves glycaemic control, cardiovascular function, body composition, and FFA in inactive middle-aged women with MetS. Practically, these results support the implementation of community-based CT programmes as an effective, non-pharmacological strategy to reduce the risk of CVD and T2DM in this high-risk population. Enhancing the accessibility and enjoyment of such programmes may also foster long-term adherence, especially among individuals who are generally less inclined to participate in structured exercise.
